# Small Intestinal Tumours

**DOI:** 10.1155/2013/702536

**Published:** 2013-11-17

**Authors:** Marcela Kopáčová, Stanislav Rejchrt, Jan Bureš, Ilja Tachecí

**Affiliations:** 2nd Department of Medicine, Charles University in Praha, Faculty of Medicine at Hradec Kralove, University Teaching Hospital, Sokolska 581, 500 05 Hradec Kralove, Czech Republic

## Abstract

*Objective*. Balloon enteroscopy (BE) and capsule enteroscopy (CE) are enteroscopy methods that allow examination and treatment of the small bowel. Before the CE and BE era, the small intestine was difficult to access for investigation. Small intestinal tumours are infrequent conditions, but about half of them are malignant. *Materials and Methods*. A total of 303 BEs were performed in 179 patients. Oral insertion was performed in 240 and anal in 63 BEs. Indications for the procedure in our patients with small bowel tumours were anaemia and/or bleeding, obstruction, suspicion of carcinoid tumour, or suspicion of Peutz-Jeghers syndrome. *Results*. In 50 of our 179 patients (28%), we diagnosed some small intestinal tumours: hamartomas in Peutz-Jeghers syndrome in 16 patients, adenocarcinoma in 7, lymphoma in 6, carcinoid tumour in 4, melanoma and stromal tumour in 3, adenoma, lipoma, and inflammatory polyps in 2, and granular cell tumour, cavernous lymphangioma, fibrolipoma, Cronkhite-Canada polyps, and metastatic involvement in individual cases. *Conclusion*. BE facilitates exploration and treatment of the small intestine. The procedure is generally safe and useful. BE and CE are essential modalities for the management of small intestinal diseases.

## 1. Introduction

The small intestine is the longest part of the digestive tract accounting for 75% of its total length and 90% of the whole mucosal surface of alimentary tract. It is not a common site for the development of neoplasms, accounting for only 3–6% of all gastrointestinal neoplasms and 1–3% of all primary gastrointestinal malignancies [1–3]. 

Multiple factors are considered to influence the relative infrequency of tumourigenesis in the small bowel: the most important are rapid small bowel transit time allowing only short contact of possible carcinogens from food with the intestinal mucosa; a high concentration of the enzymes and intestinal juices decreasing the concentration of irritating agents (benzpyrene, a known carcinogen present in food, is converted into less toxic metabolites by benzpyrene hydroxylase, which is present in higher concentrations in the small intestine compared to the stomach and colon); the high concentration of gastrointestinal lymphoid tissue and high level of immunoglobulins (IgA) exert an effective immune surveillance; a decrease in mechanical and/or chemical inflammation of the mucosa because of liquidity and alkaline pH of the small bowel contents; low bacterial load (especially anaerobic bacteria) in the small intestine processing the intestinal content produces a low amount of carcinogens; and rapid turnover of intestinal mucosa (epithelial cells) should decrease the potential growth and development of neoplastic cells [2–5]. 

The diagnosis of small intestinal tumours is difficult due to the rarity of these lesions and the nonspecific and variable nature of the presented symptoms. The most common symptom is GI bleeding, more often obscure. Apart from this, patients may be presented with nonspecific complaints such as abdominal pain, anaemia, nausea and vomiting, weight loss, malabsorption, diarrhoea, intestinal obstruction, and perforation. However, many patients are asymptomatic until the late stages of disease [5–8].

Small intestinal tumours are also difficult to identify by means of diagnostic imaging. Until recently, barium enteroclysis has been used as the best radiological possibility for detection of small bowel malignancies. Not only is the investigation time consuming and poorly tolerated by patients but it is also limited in its ability to accurately represent the mural and extramural portion of tumour and contribute to a high miss rate for small and/or flat lesions. Contemporary modern alternatives for investigation of small bowel tumours are multidetector CT and MRI techniques [5]. With the development of multislice spiral computed tomography and magnetic resonance imaging, computed tomography enteroclysis and magnetic resonance enteroclysis are widely used in the examination of the small bowel and diagnosis of small bowel tumours. Both, computed tomography and magnetic resonance enteroclysis, with three-dimensional imaging capabilities and excellent soft-tissue contrast can analyze the abnormalities of peripheral intestinal structure as well as the tunica mucosa. These two methods are even able to clearly reveal the localization, appearance, degree of mesenteric infiltration, and remote tumour metastasis, which increases our cognition of the imaging diagnosis for intestinal tumours [1, 5].

Endoscopy is the best method for investigation of the gastrointestinal tract. It has the advantage of direct visualisation of the mucosa, histology, and even therapeutic possibilities. Upper GI endoscopy performed to the ligament of Treitz is optimal for identifying duodenal tumours; colonoscopy could examine the area of the terminal ileum. However, lesions located between these points were a diagnostic challenge. Push enteroscopy is an effective diagnostic and therapeutic procedure, but only for examination of the distal duodenum and proximal jejunum to approximately 50–70 cm past the ligament of Treitz. Sonde enteroscopy was a demanding method, both for the patient and for the physician and has been completely abandoned. Intraoperative enteroscopy is the complete investigation of the small bowel with possibilities for therapy at the same time, but it is invasive and, nowadays, is reserved for multiple transmural small lesions of small intestine alone (blue rubber bleb nevus syndrome, multiple carcinoids, etc.) [9, 10]. 

The development and the introduction in the clinical practice of the capsule enteroscopy (CE) and balloon enteroscopy (BE) were a great boom in investigation of the small bowel. Both methods allowed for complete enteroscopy, in the case of BE with sampling biopsies and therapeutic potential.

## 2. Materials and Methods

A total of 303 double balloon enteroscopies (DBE) were performed in 179 patients (87 men, 92 women, mean age 48 years, range 12–86) using Fujinon EN 450T5 and EN 450P5 enteroscopes. Oral insertion was performed in 240 procedures and an anal approach was taken in 63 DBEs. Most of our patients with the anal approach primarily underwent the oral procedure. Indications for the procedure in all of our patients with small bowel tumour were anaemia and/or bleeding, obstruction, suspicion of carcinoid tumour, or suspicion of Peutz-Jeghers syndrome.

## 3. Results and Discussion

**Figure 1:**
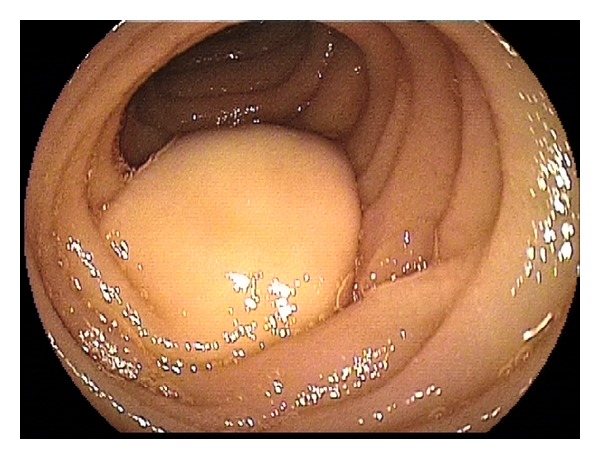
Jejunal lipoma.

**Figure 2:**
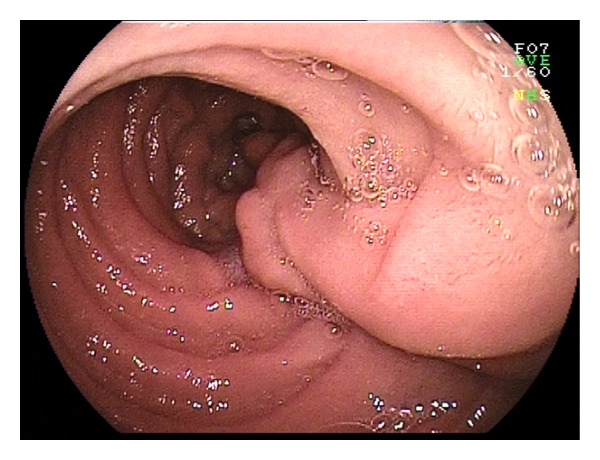
Jejunal hamartoma.

**Figure 3:**
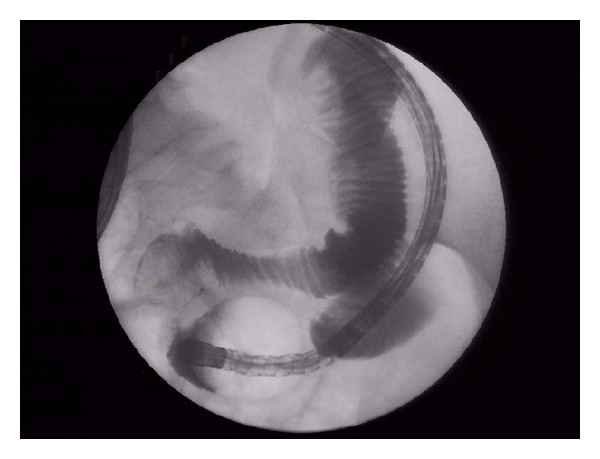
Jejunal hamartoma; the size of the polyp is nicely seen in DBE fluoroscopy.

**Figure 4:**
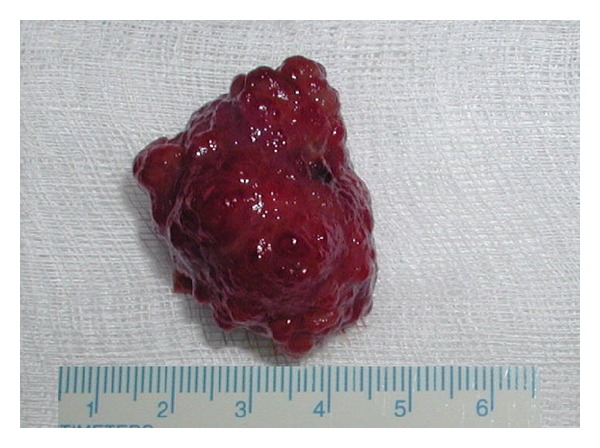
Jejunal hamartoma, resected specimen.

**Figure 5:**
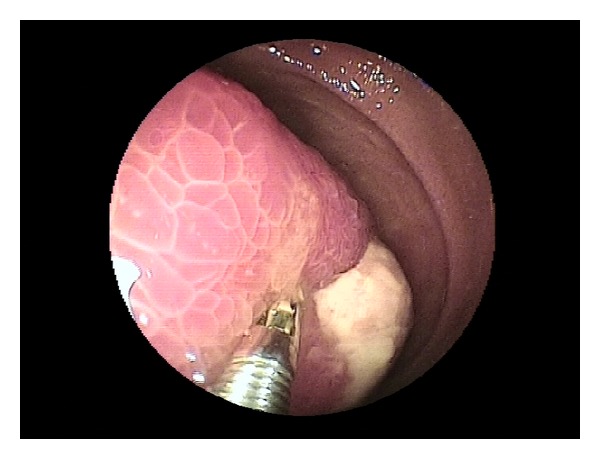
Granular cell tumour with ulcers in the ileum.

**Figure 6:**
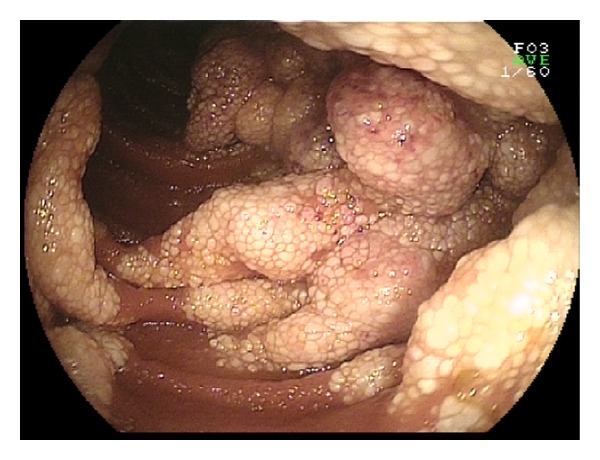
Cavernous lymphangioma of the jejunum.

**Figure 7:**
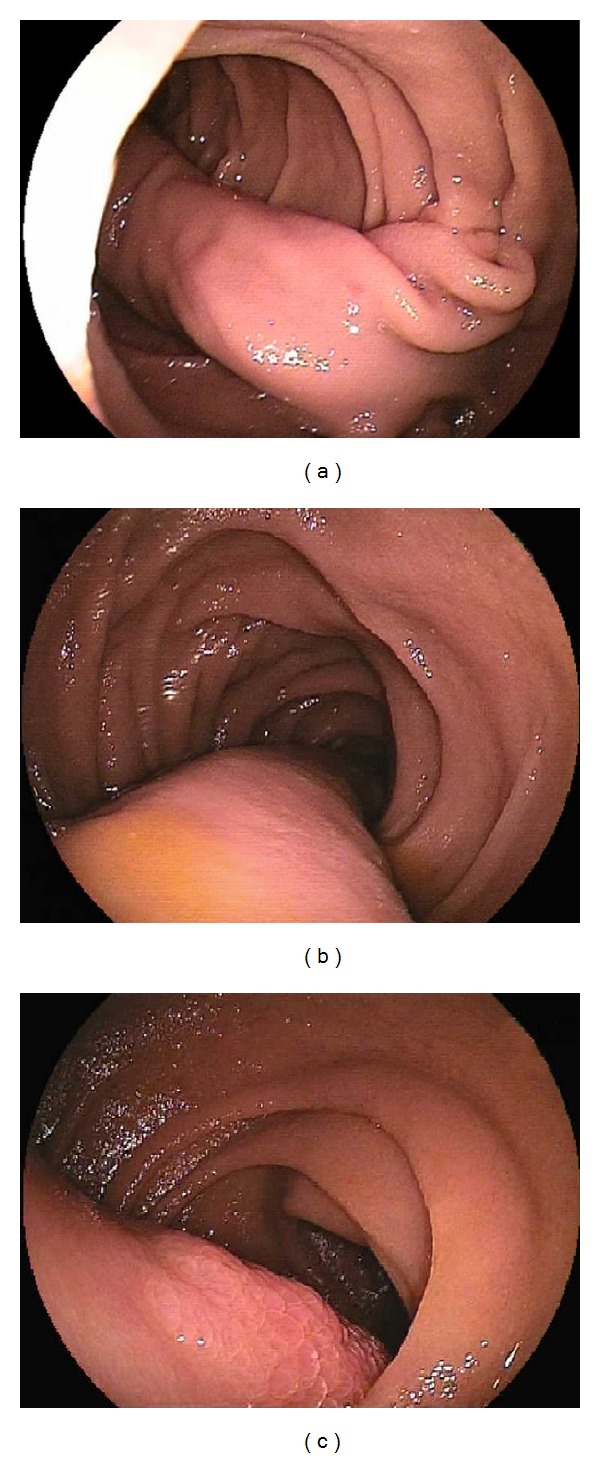
Fibrolipoma of the distals duodenum extends beyond oral jejunum ((a) stalk, (b) body, and (c) head of the polyp).

**Figure 8:**
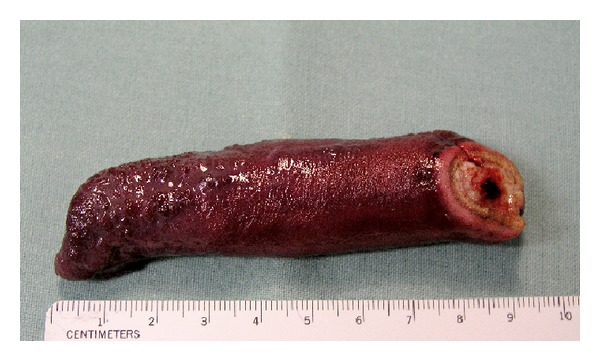
The fibrolipoma after endoscopic polypectomy.

**Figure 9:**
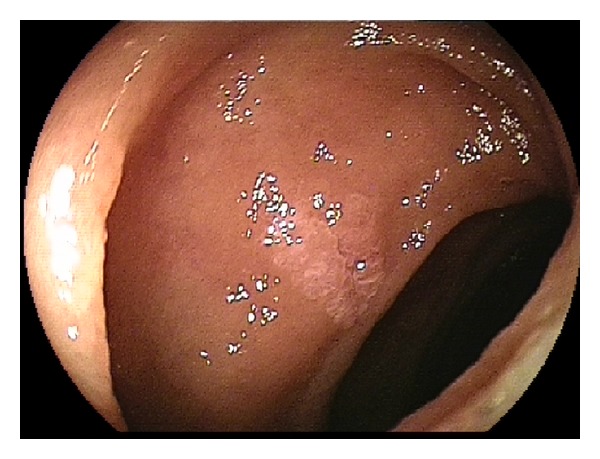
Flat adenoma of the jejunum.

We found some small bowel tumours (both, benign or malignant) in 74 of our 303 DBEs in 50 patients; 17 men, 33 women, mean age 41 years. Capsule enteroscopy preceded DBE in 21 cases and suspicion of tumour was expressed in 20 cases (one tumour was missed by capsule enteroscopy). Final diagnosis was specified using DBE in all cases. Twenty-one of our 50 patients (42%) had malignant tumours: 7 were adenocarcinomas, 6 lymphomas, 4 carcinoids, 3 melanomas, and multiple metastatic involvement of the small bowel with renal carcinoma was found once. Another 29 had benign tumours: 16 patients with Peutz-Jeghers syndrome and multiple hamartomas, 3 patients with GISTs, 2 inflammatory polyps in NSAIDs patients, 2 adenomas, 2 lipomas, 1 cavernous lymphangioma, 1 fibrolipoma, 1 granular cell tumour, and 1 time polyps in Cronkhite-Canada syndrome Figures 1–9. 

We performed 40 DBEs in our 16 patients with Peutz-Jeghers syndrome; 34 with oral and 6 with anal approach. A total number of 502 polyps were removed. One to 48 polyps were removed per session (mean 13, median 15). The largest hamartoma measured 6 cm in diameter on CT scan. 

Of our six patients with lymphoma, one suffered from T-cell lymphoma and celiac disease, one with MALT-lymphoma and Crohn's disease, one with T-cell rich B-cell lymphoma, and the last three with large B-cell lymphoma. The patient with T-cell lymphoma died within one year of diagnosis.

Until recently, diagnosis and management of small-bowel tumours were delayed by the difficulty of access to the small bowel and the poor diagnostic capabilities of the available diagnostic techniques. Early use of CE can shorten the diagnostic work-up and influence the subsequent management of these patients [7]. CE appears to be an ideal tool to recognise the presence of neoplastic lesions along this organ, since it is noninvasive and enables the entire small bowel to be visualised. High-quality images of the small-bowel mucosa may be captured and small and flat lesions recognised without exposure to radiation [3]. Recent studies on a large population of patients undergoing CE have reported small-bowel tumour frequency only slightly above that reported in previous surgical series (range 1.6%–2.4%) and have also confirmed that the main clinical indication for CE in patients with small-bowel tumours is obscure gastrointestinal bleeding. The majority of tumours identified by CE are malignant; many were unsuspected and not found by other methods. However, it remains difficult to identify pathology and tumour type based on the lesion's endoscopic appearance. Despite its limitations, CE provides crucial information leading, in most cases, to changes in subsequent patient management [3].

**Figure 10:**
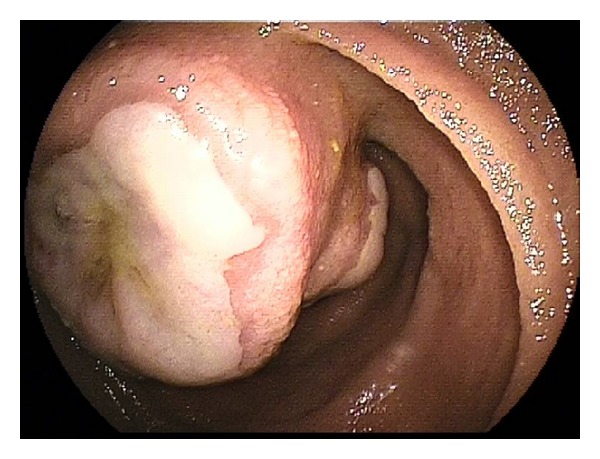
Gastrointestinal stromal tumour in the jejunum.

**Figure 11:**
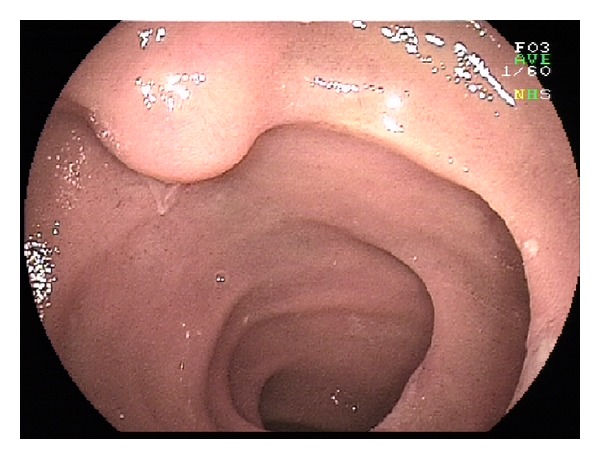
Ileal carcinoid tumour.

**Figure 12:**
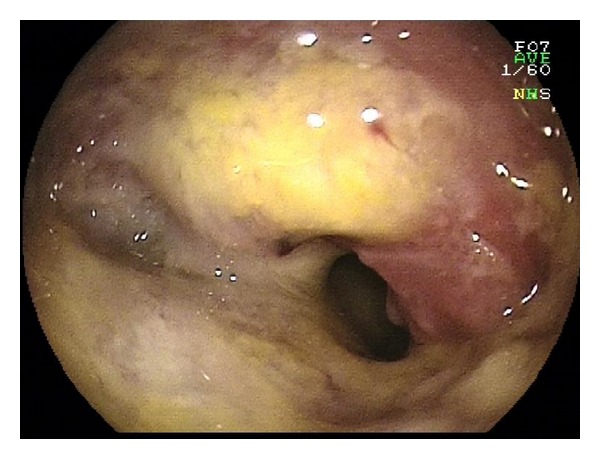
Stenosing adenocarcinoma of the jejunum.

Before the CE and BE era, the small intestine was difficult to access for endoscopic investigation and small intestinal tumours were often diagnosed as late as during surgery. A delay in diagnosis is common, which may result in the discovery of disease at a late stage. An unfortunate consequence of late diagnosis is a poor outcome of the treatment in the event of malignancies. A variety of tumours, approximately 40 different histologic types of both benign and malignant small intestinal tumours, may arise within the small intestine [6, 11, 12]. Benign lesions that may arise in the small bowel mostly include adenomas, hamartomas, leiomyomas, fibromas, and lipomas. Malignant tumours of the small intestine are very rare compared to other gastrointestinal organs but are among those with the poorest prognosis compared with other gastrointestinal malignancies [5]. It is important, that approximately half of small intestinal tumours are malignant (42% in our setting). The most common are adenocarcinomas following by lymphomas, carcinoids, and gastrointestinal stromal tumours (GISTs) Figures 10–12. The five-year survival rates range from 0–28% for adenocarcinomas, 14–30% for lymphomas, 50% for GISTs, and 60% for carcinoid tumours [5]. A small part of small intestinal malignancies comprise secondary malignancies, usually metastases of the lung, breast and prostate cancer, and malignant melanoma.

According to epidemiologic studies, the most common malignant tumours were carcinoid and adenocarcinoma, followed in order by gastrointestinal stromal tumour and malignant lymphoma. The incidence of small intestinal malignancies is increasing [13].

According to data from the USA, the distribution of histologic types of small-bowel malignant tumours is changing, largely because of the increasing incidence of carcinoids. In 1987, the most common histologic types of malignant tumours of the small intestine in population-based registry data from the Surveillance, Epidemiology and End Results (SEER) program of the National Cancer Institute were adenocarcinoma 45%, carcinoid 29%, lymphoma 16%, and sarcoma 10% [14]. Bilimoria et al. published in 2009 that carcinoid tumours surpassed adenocarcinomas as the most common small-bowel tumour (data from the National Cancer Data Base-NCDB) [15]. Between 1985 and 2005, the proportion of patients with carcinoids increased from 28 to 44 percent, while the proportion of adenocarcinoma decreased from 42 to 33 percent. The proportion of patients with stromal tumours and lymphomas remained essentially stable (17 and 8 percent, resp.) [15]. According to a large European multicentre study with capsule enteroscopy from Rondonotti et al., the main primary small-bowel tumour type was gastrointestinal stromal tumour (GIST) (32%) followed by adenocarcinoma (20%) and carcinoid (15%); 66% of secondary small-bowel tumours were melanomas [7].

Different risk factors are considered for development of small intestine neoplasms.

Certain hereditary syndromes are associated with an increased incidence of particular histologic types of small intestinal tumours. These include the Peutz-Jeghers syndrome (hamartomatous polyps occurring primarily in the jejunum and ileum), familial adenomatous polyposis and Gardner syndrome (adenoma and adenocarcinoma), and von Recklinghausen disease (paraganglioma). Desmoid tumours, which are often multiple, may be the primary manifestation of Gardner syndrome in the small bowel [12]. Peutz-Jeghers syndrome is an inherited, autosomal dominant disorder distinguished by hamartomatous polyps in the gastrointestinal tract and pigmented mucocutaneous lesions. Prevalence of this syndrome is estimated from 1 in 8,300 to 1 in 280,000 individuals. Peutz-Jeghers syndrome predisposes sufferers to various malignancies (gastrointestinal, pancreatic, lung, breast, uterine, ovarian, and testicular tumours). Bleeding, obstruction, and intussusception are common complications in patients with this disease [16]. 

Cronkhite-Canada syndrome is a sporadic disease with multiple polyps in the gastrointestinal tract. Histology finds hamartomatous polyps (juvenile type) exhibiting glandular hyperplasia, cystic dilation, mucosal edema, and eosinophilic inflammation. Extraintestinal manifestations are alopecia, dermal hyperpigmentation, onychodystrophy, diarrhoea, protein loosing enteropathy, and/or dysgeusia. Cachexia and colon cancer are most common [17].

Dietary factors have been suggested to be related to the risk of small-bowel carcinoma. A study of small-bowel carcinoma mortality and food intake by the WHO showed correlation of carcinoma with per capita daily consumption of animal fat and animal protein. Saturated fat intake seems to be a risk factor for small-bowel carcinoma. On the other hand, there is no association between alcohol intake and risk of adenocarcinoma of the small intestine [18]. There are a number of studies about obesity and small intestinal adenocarcinoma with no clear result. Men have higher incidence rates of small intestinal cancer in most countries [18].

Cigarette smoking, biliary tract diseases (cholecystitis and gallstones), infection with Helicobacter pylori, use of corticosteroids, exposure to ionising radiation, and so forth are suspect factors in small intestinal tumourigenesis [18]. 

Univocal risk factors for individual malignancies were identified. 

Adenocarcinomas are most common in the distal duodenum and the duodenojejunal junction. Explanations for the predilection to the initial part of the small bowel include the metabolism or dilution of ingested carcinogens in transit through the small bowel or interactions of carcinogens with pancreatobiliary secretions [19]. Small intestinal inflammatory disorders that predispose patients to adenocarcinoma include Crohn's disease (in these patients, tumours could be involved in 70% of the terminal ileum), celiac disease, familial adenomatous polyposis, Peutz-Jeghers syndrome, and Meckel's diverticulum [5, 15, 18]. The risk of adenocarcinoma in Crohn's disease is directly related to both the extent of small-bowel involvement and the duration of Crohn's disease. The cumulative risk of a small-bowel adenocarcinoma in patients with small bowel Crohn's disease is 0.2% at 10 years and 2.2% at 25 years of disease [20]. Typical endoscopic appearance is usually concentric tumour stenosis with irregular margins and complete bowel obstruction in the late stage. Polypoid lesions and ulcerations are sometimes seen.

Lymphoma may arise as a primary neoplasm in the intestine or as a component of systemic disease with gastrointestinal involvement. Primary intestinal lymphomas arise from the lymphoid aggregates in the submucosa. The distribution of lymphomas in the small intestine parallels the distribution of lymphoid follicles, with the lymphoid rich ileum representing the most common location. The diagnosis of primary gastrointestinal lymphoma is characterised by no peripheral or mediastinal lymphadenopathy, normal white and differential blood cell count, and no evidence of liver or spleen involvement [21]. Primary gastrointestinal lymphoma is the most common extranodal form of lymphoma; the stomach and small bowel are the most common sites. A typical endoscopic appearance is polypoid mass with ulcerations and bowel obstruction in the late stage. Bleeding is quite a common sign, and patients are at risk of spontaneous small-bowel perforation. Small-bowel lymphomas are more often found in patients with celiac disease, Crohn's disease, AIDS, EBV infection, immunoproliferative disease (IPSID), long term immunosuppressive therapy (posttransplantation) and after radiation, and/or chemotherapy [5, 21]. A significant increase in the incidence of EATL in the United States was indicated, which may reflect the increasing seroprevalence of celiac disease and better recognition of rare types of T-cell lymphomas [22]. EATL is a rare disease with an incidence of 0.1 per 100,000 inhabitants per year, occurring in older age, with a peak incidence in the 7th decade, and nonelevated during childhood and adolescence. Small intestinal lymphomas and carcinoma are responsible for elevated morality risk in celiac disease. Although uncomplicated celiac disease is twice as frequent in female patients, EATL is more prevalent in males [23–25].

Carcinoids, gastroenteropancreatic neuroendocrine tumours, constitute a diverse group of neoplasms arising from the diffuse neuroendocrine cell system. During the last 2 years, a new classification system, the WHO 2010, has come into clinical practice together with Tumour Nodes Metastases (TNM) staging and grading systems, developed by the European Neuroendocrine Tumour Society/American Joint Cancer Committee [26]. 

Carcinoid is most commonly found in the ileum within 60 cm of the ileocecal valve; duodenal or jejunal localisation is rare. Small-bowel carcinoids are likely to be associated with MEN 1. Endoscopically, a small submucosal protrusion with normal mucosa on the surface is usually seen. Desmoplastic reaction in submucosa is common.

Gastrointestinal stromal tumour (GIST) could be found elsewhere in the small intestine, but ileal localisation is more common. A regular round mass with normal mucosa on its surface and often central ulceration with or without visible bleeding is found on endoscopy. Large vessels around the tumour are often seen during surgery.

## 4. Conclusion

Small intestinal tumours are rare diseases, but approximately half of them are malignant. A lack of symptoms and difficult determination lead to late diagnosis with advanced disease and poor prognosis. Because of nonspecific symptoms, it is difficult to make a diagnostic algorithm. Nowadays, the best way to ensure earlier diagnosis seems to be the use of CE in combination with other new diagnostic (MRI or multidetector CT enterography) and therapeutic endoscopic (BE) techniques. These tools could lead to earlier diagnosis and treatment of these neoplasms, better survival rate, and cost savings in the end.

Prevention in known risk factors and chronic diseases is obvious.
